# Identification and Validation of Oncologic miRNA Biomarkers for Luminal A-like Breast Cancer

**DOI:** 10.1371/journal.pone.0087032

**Published:** 2014-01-31

**Authors:** Ailbhe M. McDermott, Nicola Miller, Deirdre Wall, Lorcan M. Martyn, Graham Ball, Karl J. Sweeney, Michael J. Kerin

**Affiliations:** 1 Discipline of Surgery, School of Medicine, National University of Ireland, Galway, Ireland; 2 School of Mathematics, Statistics and Applied Mathematics, National University of Ireland, Galway, Ireland; 3 School of Science and Technology, Nottingham Trent University, Nottingham, United Kingdom; University of Salerno, Italy

## Abstract

**Introduction:**

Breast cancer is a common disease with distinct tumor subtypes phenotypically characterized by ER and HER2/*neu* receptor status. MiRNAs play regulatory roles in tumor initiation and progression, and altered miRNA expression has been demonstrated in a variety of cancer states presenting the potential for exploitation as cancer biomarkers. Blood provides an excellent medium for biomarker discovery. This study investigated systemic miRNAs differentially expressed in Luminal A-like (ER+PR+HER2/*neu*-) breast cancer and their effectiveness as oncologic biomarkers in the clinical setting.

**Methods:**

Blood samples were prospectively collected from patients with Luminal A-like breast cancer (n = 54) and controls (n = 56). RNA was extracted, reverse transcribed and subjected to microarray analysis (n = 10 Luminal A-like; n = 10 Control). Differentially expressed miRNAs were identified by artificial neural network (ANN) data-mining algorithms. Expression of specific miRNAs was validated by RQ-PCR (n = 44 Luminal A; n = 46 Control) and potential relationships between circulating miRNA levels and clinicopathological features of breast cancer were investigated.

**Results:**

Microarray analysis identified 76 differentially expressed miRNAs. ANN revealed 10 miRNAs for further analysis (*miR-19b, miR-29a, miR-93, miR-181a, miR-182, miR-223, miR-301a, miR-423-5p, miR-486-5* and *miR-652*). The biomarker potential of 4 miRNAs (*miR-29a, miR-181a*, *miR-223* and *miR-652*) was confirmed by RQ-PCR, with significantly reduced expression in blood of women with Luminal A-like breast tumors compared to healthy controls (p = 0.001, 0.004, 0.009 and 0.004 respectively). Binary logistic regression confirmed that combination of 3 of these miRNAs (*miR-29a, miR-181a* and *miR-652*) could reliably differentiate between cancers and controls with an AUC of 0.80.

**Conclusion:**

This study provides insight into the underlying molecular portrait of Luminal A-like breast cancer subtype. From an initial 76 miRNAs, 4 were validated with altered expression in the blood of women with Luminal A-like breast cancer. The expression profiles of these 3 miRNAs, in combination with mammography, has potential to facilitate accurate subtype-specific breast tumor detection.

## Introduction

Breast cancer is a prevalent disease, accounting for significant morbidity and mortality with a worldwide incidence of over 1,300,000 women [1]. It is the commonest female malignancy in almost all European countries and in North America and leading cause of female cancer mortality. Breast cancer is a heterogeneous disease, with distinct tumor phenotypes reflecting a spectrum of underlying molecular alterations and initiating events [2]. Analysis of gene expression patterns governing these events has resulted in the classification of breast tumors into subtypes broadly determined by expression of the estrogen receptor (ER), progesterone receptor (PR) and human epidermal growth factor receptor (HER2/*neu*). Targeted therapies including hormonal therapy for ER positive tumors and trastuzumab to inhibit HER2/*neu* signaling have become the major components of adjuvant breast cancer management. Consequently, when diagnosed and treated early, breast cancer is highly curable. Despite these advances, hematogenous spread of malignant cells from the primary tumor to distant organs with subsequent proliferation into metastases remains the leading cause of death for breast cancer patients [3]. Further insight into the molecular mechanisms underlying tumorigenic transformation is clearly warranted for the identification of additional molecular predictors and disease biomarkers in the clinical management of breast cancer.

Much current cancer research is focused on the identification of circulating cancer-specific biomarkers for application to disease diagnostics, as well as predicting and monitoring response to disease and tumor recurrence. There are no reliable circulating biomarkers for breast cancer. Mammography is the most widespread screening tool, with a definitive diagnosis requiring an invasive tissue biopsy. This prevalent disease is in need of a minimally invasive biomarker which may be used in combination with radiological imaging to facilitate early subtype specific tumor diagnosis. Blood presents an excellent medium for biomarker discovery; it is minimally invasive and simple to obtain during routine clinical examination. Moreover, blood circulates throughout the body delivering nutrients and carrying proteins (including miRNAs), hormones and cells while eliminating waste substances, thereby reflecting the summation of physiological and pathological processes occurring in an individual at any one time.

Mi(cro)RNAs have shown much potential as cancer-specific biomarkers. MiRNAs regulate gene expression at the post-transcriptional level and are intimately linked with the cancer state; Firstly, miRNA expression has a causal effect on tumourigenesis, acting as oncogenes and tumor suppressor genes and secondly, altered miRNA expression occurs as a result of the carcinogenic process. In breast cancer, altered tissue miRNA expression patterns have been shown to correlate with molecular subtype and hormonal receptor status [4], [5]. MiRNAs were originally studied in tissue, but several studies have demonstrated that tumor-specific miRNAs are detectable in the circulation [6]–[8]. These studies allude to the promising role of circulating miRNAs as biomarkers for detection of disease. Furthermore, speculation that circulating miRNA profiles could reflect not only the tumor tissue-type, but also the intrinsic molecular subtype thus acting as a fluid biopsy would be particularly valuable in breast cancer where management, even immediately following diagnosis, is governed by hormonal and HER2/*neu* receptor status, largely conveying molecular subtype.

Luminal A is the most common subtype, including over 70% of breast cancers. Confirmation of Luminal A subtype is performed using mRNA expression analysis however phenotypically Luminal A-like tumors are characterized as hormone receptor positive and HER2/*neu* negative. These tumors are frequently screen detected, node negative and therefore associated with a good prognosis. Recent advances such as the development of the Oncotype DX® test strive to prevent overtreatment of this common subtype by identifying women at high risk of recurrence for adjuvant chemotherapy.

The aim of this study was to utilize microarray profiling to identify circulating miRNAs that are differentially expressed in women with Luminal A-like breast cancer (ER positive, PR positive, HER2/*neu* negative) in comparison to healthy controls, to validate candidate miRNA expression using RQ-PCR, investigate their expression level in association with common clinicopathological parameters, and to study their effectiveness as circulating diagnostic biomarkers in the clinical setting.

## Methods

### Study Cohort and Sample Collection

Blood samples were prospectively collected from 110 women; this included 54 consecutive patients with a new diagnosis of Luminal A-like breast cancer and 56 healthy control participants. All patients had histologically confirmed Luminal A-like breast cancer; Hormone receptor positive and HER2/*neu* negative. Definitive confirmation of Luminal A subgroup would have required mRNA expression profiling which was not routinely performed or available at our institution. Healthy control blood samples were collected from women residing in the same catchment area as the cancer cases. These women were interviewed by a clinician in advance of sample collection to ensure that there was no personal history of malignancy or current inflammatory or infectious condition. Venous non-fasting whole blood samples were collected in BD vacutainers® containing 18 mg dipotassium EDTA anticoagulant (BD-Plymouth, PL6 7BP, UK). Microarray profiling was performed on RNA derived from blood on 10 of the above patients and 10 controls, the clinicopathological details of which are presented in Table 1. The remaining 44 cases and 46 controls were used to independently validate microarray findings. Clinicopathological details of the validation group are shown in Table 2. Tissue specimens both tumor (n = 11) and tumor-associated normal (TAN, n = 10) were prospectively collected from patients with Luminal A-like breast cancer at the time of surgical resection. Tissue samples were collected in RNAlater® RNA stabilization reagent (Qiagen, UK) prior to cryopreservation at −80°C. Clinicopathological details of this cohort are included in Table 3.

**Table 1:** Clinicopathological patient data for blood samples analyzed by microarray.

Cases	Age (yrs)	Inv. T size(mm)	Whole Tsize (mm)	HistologicalSubtype	NodalStatus	Grade	UICCStage	ER	PR	HER2/neu	Controls*	Age (yrs)
1	70	22	22	Inv. Muc.	–	2	1	+	+	–	1	81
2	52	15	15	Inv. Ductal	–	2	2	+	+	–	2	61
3	60	13	20	Inv. Ductal	–	2	2	+	+	–	3	82
4	50	22	22	Inv. Ductal	–	2	1	+	+	–	4	76
5	46	38	38	Inv. Ductal	+	1	2	+	+	–	5	74
6	59	120	120	Inv. Ductal	+	2	3	+	+	–	6	87
7	56	53	53	Inv. Lobular	+	2	2	+	+	–	7	94
8	55	61	61	Inv. Ductal	+	2	3	+	+	–	8	94
9	44	45	45	Inv. Ductal	–	2	2	+	+	–	9	96
10	75	50	60	Inv. Ductal	+	2	3	+	+	–	10	72

Yrs, Year; mm,Millimeters; Inv. T size, Invasive Tumor size; UICC, Stage of breast tumor according to the International Union Against Cancer staging criteria; ER, Estrogen receptor; PR, Progesterone receptor: HER2/*neu*, Human epidermal growth factor receptor; –, negative; +, positive; NA not applicable. *All control subjects had no personal or family history of breast or ovarian cancer and were clinically well at the time of sampling.

Luminal A-like is phenotypically defined as ER positive, PR positive, HER2/neu negative.

**Table 2:** Clinicopathological Patient Data for Blood in Independent Validation Cohort.

Luminal A Breast Tumors				Number (%) 44
Mean age, years (range)				59.86 (±13.45)
Median Whole. T size (mm)				32.82 (±26.30)
			Missing data	11
Median Inv. T size (mm)				27.41 (±20.74)
			Missing data	22
Histological Subtype				
			Invasive ductal	35
			Invasive lobular	1
			Other	7
			Missing	1
Nodal status				
			Positive	22
			Negative	19
			Missing	3
Grade				
			1	7
			2	32
			3	4
			Missing	1
UICC stage				
		Stage 1		15
		Stage 2		14
		Stage 3		8
		Stage 4		2
		Missing		3
Estrogen Receptor				
		Positive		44 (100%)
		Negative		0
Progesterone Receptor				
	Positive			44 (100%)
	Negative			0
HER2/*neu* Receptor				
	Positive			0
	Negative			44 (100%)
Subtype				
	Luminal A-like			44 (100%)
**Controls**				**46**
Mean Age, years (range)				44.21 (±20.61)

**Table 3:** Clinicopathological Patient Data for Breast Tumors.

Luminal A Breast Tumors		Number (%) 11
Mean age, years (range)		54.27 (±9.26)
Median Whole. T size (mm)		34.1 (±42.5)
Histological Subtype		
	Invasive ductal	11
Grade		
	1	1
	2	1
	3	8
	Missing	1
UICC stage		
	Stage 1	3
	Stage 2	8
Estrogen Receptor		
	Positive	11 (100%)
	Negative	0
Progesterone Receptor		
	Positive	11(100%)
	Negative	0
HER2/*neu* Receptor		
	Positive	0
	Negative	11 (100%)
Subtype		
	Luminal A-like	11 (100%)

### Ethics Statement

Ethical approval was granted by the Clinical Research Ethics Committee, Galway University Hospital. Written informed consent was obtained from all study participants.

### RNA Extraction

Total RNA was extracted from 1 ml of blood using TRI Reagent BD (Molecular Research Centre, Inc) as previously described [9]. RNA concentration and integrity were examined by NanoDrop spectrophotometry (NanoDrop ND-1000 Technologies Inc., DE, USA) and Agilent Bioanalyzer RNA 6000 NanoChip Kit Series II (Agilent Technologies, Germany) analysis, respectively.

### MiRNA Microarray Profiling

Expression profiling of circulating miRNAs was performed for 20 samples as described above using TaqMan human miRNA arrays and assays in accordance with the manufacturer’s instructions (Taqman Low Density Array Human microRNA, Applied Biosystems, Foster City, CA, USA). In brief, total RNA was reverse transcribed using Megaplex primer pool A (Applied Biosystems) which contained sequence-specific primers for 381 specific miRNAs plus 3 controls (pool A). An additional panel of 384 miRNAs (381 miRNAs and 3 controls, pool B) was performed on a subset of 4 cancers and 4 controls. Real-time quantitative PCR was performed for 667 miRNAs, using A and B microfluidic cards, each containing primers and probes for 381 specific miRNAs plus 3 controls and thermal-cycled on an Applied Biosystems 7900 HT instrument. MiRNA expression data are available from the National Center for Biotechnology Gene Expression Omnibus (GEO) at accession number GSE46355.

### Microarray Data Analysis

Within this study normalized miRNA array data were analyzed within a nonlinear ANN based data mining algorithm to identify those with altered expression in Luminal A-like breast cancer. This method comprised a feed-forward back-propagation algorithm utilizing a three layer architecture, a sigmoid transfer function, 2 hidden nodes and early stopping on unseen data full details are described by Lancashire *et al* [10]). Monte Carlo Cross validation was applied to the modeling approach to determine the performance of the miRNA probes on a randomly selected blind subset. This approach addressed issues with false discovery by preventing over fitting, driving the solution to one that has good predictability for a blind population.

**Figure 1:**
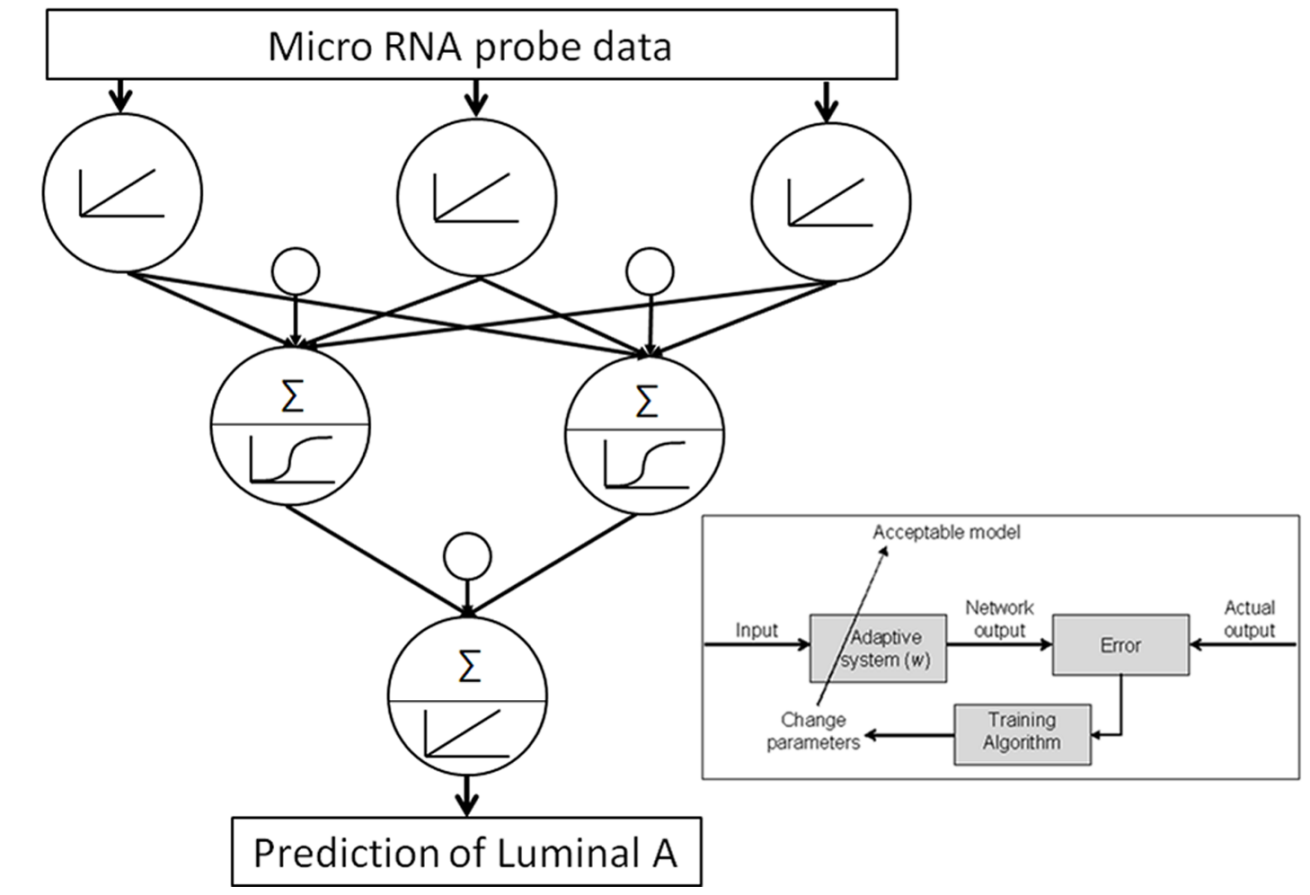
ANN architecture and algorithm as applied to data mining for miRNA markers of Luminal A-like breast cancer.

The performance of single miRNA probes was determined by developing ANN models using the algorithm described above (each using a single probe intensity from the data), to classify between Luminal A-like breast cancer and healthy controls (Figure 1). This process was repeated for all of the probes on the array and their classification performance on blind data determined. In this way a rank order of miRNAs was determined. From this rank order the key miRNAs were taken forward for validation.

### Validation by RQ-PCR

Quantification of individual miRNAs in both blood and tissue samples was determined by RQ-PCR using TaqMan miRNA assays (Applied Biosystems). Ten of the most differentially expressed miRNAs from the microarray screen were selected for validation. Following RNA isolation, 100 ng of total RNA was reverse transcribed using stem-loop primers and MultiScribe reverse transcriptase. PCR reactions were performed in triplicate in final volumes of 10 µl on 96 well plates. Each plate included an inter assay control (IAC) to account for run-to-run variation. Plates were run on a 7900 HT instrument (Applied Biosystems) using standard thermal-cycling conditions.

Raw fluorescence (cycle threshold, C_T_) data were subsequently calculated. High C_T_ values indicated low miRNA expression and vice versa. The threshold standard deviation for intra- and inter-assay replicates was 0.28. PCR amplification efficiencies (E) were calculated for each miRNA and Taqman miRNA assay using the formula E = (10−1/slope−1)×100, using the slope of the semi-log regression plot of Ct versus log input of cDNA (10-fold dilution series of five points). A threshold of 10% above or below 100% was adopted. C_T_ values were scaled to lowest expressing sample and normalized to *miR-16,* which has been shown to be stably expressed in breast cancer and is the most widely used endogenous control miRNA for breast cancer thus far [9], [11]. MiRNA expression was calculated by the comparative cycle threshold (ΔC_T_) method, using qbase^PLUS^® software (Biogazelle, NV, Belgium).

### Statistical Analysis

Statistical analysis was performed using Minitab version 16.0 (Minitab Ltd, Coventry UK). The Kolmogorov-Smirnov test for normality was conducted. Data were log transformed (log_10_) for analysis when non-normal distribution was identified. Significance and associations of circulating miRNA levels were determined using the Mann-Whitney U test, t-test, ANOVA, Spearman’s Rho or Pearson correlation, as appropriate. Results with p-value less than 0.05 were deemed to be significant. Binary logistic regression analysis was used and receiver operating characteristic (ROC) curves were generated to evaluate the ability of chosen miRNAs to distinguish between cancer cases and controls. This was performed both individually and for combinations of miRNAs.

## Results

### Identification of Dysregulated miRNAs in Luminal A-like Breast Cancer

The ANN data mining algorithm identified 76 miRNAs with detectable and altered expression in blood of patients with Luminal A-like breast cancer compared to healthy controls (Table S1).

### Validation of Microarray

**Figure 2:**
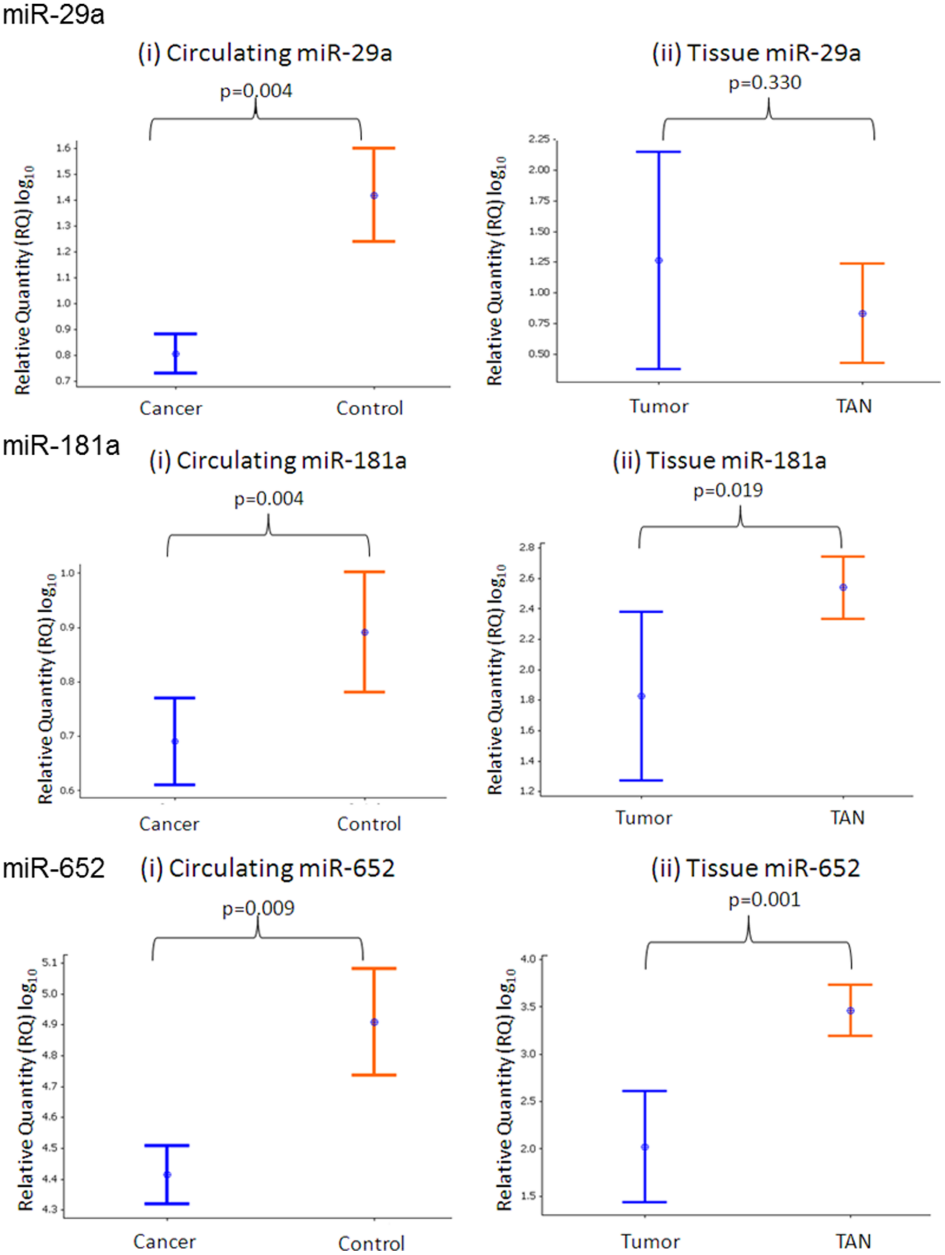
Expression levels of (A) *miR-29a* (i) in the circulation of patients with Luminal A-like breast cancer versus healthy controls and (ii) *miR-29a* levels in tumour and associated normal (TAN) tissue; (B) *miR-181a* expression (i) in the circulation of cases and controls and (ii) and in tumour and TAN tissue; (C) *miR-652* expression in the circulation of cases and controls (i) and in tumour and TAN tissue (ii).

To further evaluate the expression patterns of individual miRNAs derived from the microarray dataset, real-time quantitative PCR was performed. A subset of three candidate miRNAs was chosen for sample to sample expression analysis and in most cases revealed good correlation between the microarray profiling data and RQ-PCR validation (Figure S1). Expression of ten of the most deregulated miRNAs was confirmed in an independent cohort of blood from patients with luminal A-like breast cancer (n = 44) and healthy controls (n = 46). MiRNA expression levels were also measured in tumor tissue derived from patients with Luminal A-like breast tissue. The miRNAs selected for validation and results obtained are outlined in Table 4. Two miRNAs (*miR-181a* and *miR-652*) were found to be over-expressed in the microarray and were down-regulated in the circulation of women with Luminal A-like breast tumors in the validation group (p = 0.004, and 0.009, respectively, Figure 2). Both *miR-181a* and *miR-652* miRNAs were also under-expressed in Luminal A-like tumor tissue compared to TAN (p = 0.019 and p<0.001, respectively Figure 2). *MiR-29a* and *miR-223* were under-expressed in the circulation of those with Luminal A-like breast cancer compared to healthy controls, in both the array and the validation cohorts (p<0.001 and p = 0.004, Figure 2).

**Table 4:** Candidate miRNAs for validation by RQ-PCR.

miRNA	Chromosomal Location	Sequence	Expression	p-value (Circulation, RQ-PCR) *	p-value (Tissue, RQ-PCR)*
miR-19b	Chromosome 13:92001446–92005532 (+)	UGUGCAAAUCCAUGCAAAACUGA	Unchanged	0.775	NA
**miR-29a**	Chromosome 7:130561506–130561569 [−]	UAGCACCAUCUGAAAUCGGUUA	**Down**	**0.001**	**0.330**
miR-93	Chromosome 7:99691391–99691470 [−]	CAAAGUGCUGUUCGUGCAGGUAG	Unchanged	0.399	NA
**miR-181a**	Chromosome 1:198828173–198828282 [−]	AACAUUCAACGCUGUCGGUGAGU	**Down**	**0.004**	**0.019**
miR-182	Chromosome 7:129410223–129410332 [−]	UUUGGCAAUGGUAGAACUCACACU	Unchanged	0.355	NA
**miR-223**	Chromosome X:65238712–65238821 [+]	UGUCAGUUUGUCAAAUACCCCA	**Down**	**0.004**	**NA**
miR-301a	Chromosome 17:57228497–57228582 [−]	CAGUGCAAUAGUAUUGUCAAAGC	Unchanged	0.179	NA
miR-423-5p	Chromosome 17:28444097–28444190 [+]	UGAGGGGCAGAGAGCGAGACUUU	Unchanged	0.519	NA
miR-486-5p	Chromosome 8:41517959–41518026 [−]	UCCUGUACUGAGCUGCCCCGAG	Unchanged	0.333	NA
**miR-652**	Chromosome X:109298557–109298654 [+]	AAUGGCGCCACUAGGGUUGUG	**Down**	**0.009**	**0.001**

Table Legend: *p-value determined by t-test.

### Association/Relationship between miRNA Expression and Clinicopathological Parameters

MiRNA (*miR-29a, miR-181a* and *miR-652*) expression data was compared with clinicopathological variables, namely grade, nodal status, tumor size and stage of disease. *MiR-29a*, *miR-181a* and *miR-652* were significantly down-regulated in the blood of patients compared to controls, irrespective of tumor grade, nodal status or stage of disease (Table 5). Altered expression in both early and late stage disease is an important biomarker characteristic. Interestingly, *miR-181a* was significantly down-regulated in the blood of patients with node positive disease compared to healthy controls (p = 0.006) but not node negative disease (p = 0.09). There was no difference in *miR-181a* expression between node positive or node negative disease. There was a negative correlation between *miR-181a* expression and invasive tumor size (Pearson correlation coefficient r = −429, p = 0.059).

**Table 5:** miRNA expression and clinicopathological parameters.

Clinicopathological Parameter	miRNA	P-value*
Stage	*miR-29a*	0.737
	*miR-181a*	0.058
	*miR-652*	0.511
Grade	*miR-29a*	0.193
	*miR-181a*	0.924
	*miR-652*	0.998
Nodal Status	*miR-29a*	0.845
	*miR-181a*	0.257
	*miR-652*	0.845

This table demonstrates that although *miR-29a*, *miR-181a* and *miR-652* are under-expressed in women with Luminal A-like breast tumors, there is no significant difference in miRNA expression levels in the blood of women with breast cancer, regardless of stage of disease (1 to 5), grade of disease (1 to 3) or nodal status (positive or negative). This is an important biomarker trait, as it reflects miRNA expression alteration in early, as well as late stage disease.

* p-value determined by one-way ANOVA.

### Biomarker Potential of miRNAs

**Figure 3:**
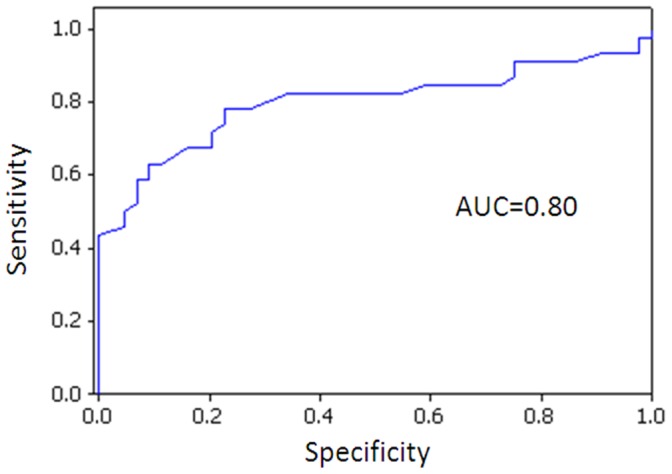
Receiver Operator Characteristic (ROC) Curve for 3 miRNA combination (*miR-29a, miR-181a* and *miR-652*).

The evident dysregulation of *miR-29, miR-181a* and *miR-652* in the blood of women with Luminal A-like breast cancer, irrespective of tumor stage or grade, revealed a potential role for these miRNAs as circulating biomarkers for Luminal A-like breast cancer detection. We compared the area under the curve (AUC) produced from receiver operator characteristic (ROC) curve generation using binary logistic regression analysis for each individual miRNA and miRNA combination profiles. The best AUC cut-off of 0.80 was generated from a combination of *miR-29a*, *miR-181a* and *miR-652*, providing a sensitivity and specificity of 77% and 74%, respectively (Figure 3). The addition of *miR-223* did not improve the sensitivity or specificity profile achieved.

## Discussion

Mammography is currently the gold standard screening tool for breast cancer diagnosis; however accurate diagnosis and intrinsic subtype confirmation requires histological evaluation from tissue obtained at breast biopsy, an invasive procedure. The identification of novel reliable minimally invasive breast cancer biomarkers would represent a significant development in the clinical management of this complex disease. The concept of a panel or profile of miRNAs for diagnostic purposes is a realistic approach, as to date no single miRNA has been reported with the qualities (sensitivity, specificity and reproducibility) for use in isolation. The 3 miRNAs identified in this study yielded a sensitivity and specificity of 77% and 74% respectively, and could be evaluated from blood collected during a simple blood test. Although not perfect, this sensitivity and specificity profile exceeds that of several currently used clinical biomarkers [12]–[15] and could be improved with the combination of mammography. There is no routinely used circulating biomarker for breast cancer detection. Carcinoma Antigen 15-3 (CA 15-3) and Carcinoembryonic Antigen (CEA) are circulating biomarkers. However their clinical application in breast cancer management is, if any, confined to detecting and monitoring disease recurrence and progression. These markers are merely elevated in 10% of stage 1 disease and 20% of stage 2 disease, precluding any usefulness in the diagnostic arena.

Early miRNA-related research mainly focused on tissue, with several reports of aberrant miRNA expression in breast cancer correlating with clinico-pathological variables such as stage and hormone receptor status [5], [16]–[20]. Furthermore, individual miRNAs have been associated with metastatic potential of breast tumors [21]. The rush to identify non-invasive diagnostic biomarkers for breast cancer has resulted in a surge of interest in circulating miRNAs. Several studies to date have evaluated miRNA expression in blood of women with breast cancer [11], [22]. Not all reports in the literature are directly comparable, as although circulating miRNAs are analyzed in each case, three alternative blood components have been used, namely whole blood, serum and plasma. We chose to analyze whole blood in this study as stability of miRNAs in EDTA-whole blood and the potential to profile miRNAs from this medium have been demonstrated [11], [23], [6]. In addition, given that circulating miRNA research is still in its infancy, it was chosen to utilize methods that could potentially be exploited in larger multi-centric trials by collecting whole blood stored in a refrigerator until transport rather than plasma or serum that requires prompt centrifugation, alloquotting and freezing.

It has been suggested that circulating miRNAs may reflect the presence of breast tumors but not the specific profiles of miRNAs within the breast tumors [24], [25]. In the current study, we identified four miRNAs (*miR-29a*, *miR-181, miR-223* and *miR-652*) with dysregulated expression in the circulation of women with Luminal A-like breast cancer. *MiR-181a* and *miR-652* were down-regulated in Luminal A-like breast tumor tissue, while *miR-29a* was not. These findings support the hypothesis that circulating miRNA expression profiles may not act as a direct window on tumor activity and brings into question the mechanism by which they enter the blood stream, in addition to their functional role, if any, in the peripheral circulation. These processes remain poorly understood. MiRNAs can enter the peripheral circulation following selective secretion from tumor cells or circulating micro-vesicles [26]. Other cells in the tumor microenvironment can also secrete miRNAs. Meanwhile another school of thought suggests that miRNAs may be detectable in the circulation as a consequence of passive leakage from apoptotic and necrotic cells [27]. In reality it is likely that both of these theories are true, with accumulating evidence to support both plausible proposals.

Once in the circulation, miRNA transport is not uniform. Some miRNAs are encapsulated in microvesicles, apoptotic bodies, exosomes or high-density lipoprotein (HDL) particles while others are in combination with proteins of the Argonaute (AGO) family [28]–[30]. The protection conveyed by microparticles or in combination with AGO proteins explains the stability of miRNAs in nuclease rich and protease rich environments, such as the circulation, when compared to mRNA [31]. The majority of circulating miRNAs, as much as 90–95%, are transported in combination with the AGO protein family [30], [31]. The functional role of miRNAs in circulation has yet to be fully elucidated; are these tiny particles merely secreted as by-products of physiological and pathological processes or are they circulating messengers, with important intercellular and inter-organ cell to cell messaging capabilities? Some recent studies allude to the potential for exosomally-packaged miRNA to act as cell to cell signaling molecules, during viral infection, the immune response and most significantly cancer progression [32]–[34]. However, despite these reports, it is likely that the majority of circulating extracellular miRNAs, particularly the AGO-transported form, have no functional role. Nonetheless, regardless of their source, their presence, relative stability and ease of detection can be exploited for biomarker means.

In this study ANN identified four specific miRNAs as being significantly altered in the circulation of women with Luminal A-like breast cancer. ANN data-mining algorithms have been shown to provide a robust solution to issues encountered within miRNA array data [5]. They have been shown to cope with non-linearity, and complexity; whilst offering the ability to identify biomarkers of high biological relevance and good predictive sensitivity and specificity [10]. *MiR-181a* has previously been reported as being significantly under-expressed in the serum of women with breast cancer compared to healthy controls [35]. It has also been shown to be downregulated in tumor tissue of lung, oral, hepatocellular, and ovarian cancers [36]–[39]. In addition, *miR-181a* was identified as a potential prognostic factor for colorectal and gastric cancer [40], [41]. A recent study, using NGS-SOLiD sequencing followed by validation with RQ-PCR reported *miR-29a* as being over-expressed in the serum of women with breast cancer [42]. This miRNA has been implicated in other cancers, predominantly colorectal where it may have a role in prognostication [43], [44]. *MiR-223* has been reported in serum of patients with nasopharyngeal carcinoma and gastric cancer [45], [46]. *In vitro* analysis revealed that *miR-223* was detected within exosomes and increased invasiveness of co-cultured cell lines (SKBR2 and MDA-MD-321) [47]. In the present study, validation of *miR-223* expression was examined in fewer samples than were available for *miR181a*, *miR29a* and *miR-652* validation (29 cancers, 40 controls), however we found it to be significantly lower in the circulation of cancer patients, *p* = 0.004). There are no previous reports, to our knowledge, of a role for *miR-652* as a diagnostic biomarker for breast cancer.

Despite the rapidly evolving field of circulating miRNAs as oncologic biomarkers, there are still a number of challenges which much be overcome before miRNA profiling can be routinely incorporated into the diagnostic arena. Real time is the most common technique employed for miRNA quantification. Despite significant technological advances in PCR instrumentation, and levels of detection, there remains little consensus on assay design through to data analysis. In particular, there is a lack of concordance on protocols for data normalization.

Although these results are extremely promising, and substantiate the potential application of miRNAs as biomarkers for breast cancer, we recognize that this study has limitations. The sample size is relatively small; larger validation analyses, involving blinded samples are needed to confirm the clinical utility of the 3 miRNA panel for luminal A-like breast cancer detection. Such studies should ideally include blood samples from all breast tumor subtypes, namely Luminal B, HER2/*neu* over-expressing and basal subgroups, as well as from patients with benign breast disease. Future studies to evaluate the mechanism of action of these miRNAs, if any, in breast tumors and determine the exact processes by which *miR-29a, miR-181a, miR-223* and *miR-652* are shed into the circulation are also warranted.

The potential value of the miRNAs outlined in this study is not restricted to diagnostic biomarkers for breast cancer. The realm of miRNA-related therapeutic strategies is gaining increased momentum, particularly in hepatitis and hepatocellular carcinoma. MiRNAs with depleted expression levels may be restored to ‘normal’ levels by viral vector encoded miRNAs or miRNA mimetics. It seems plausible if these miRNAs have a functional role in the tumor microenvironment, tumourigenesis could potentially be halted or reversed by restoring their expression levels.

## Conclusions

In conclusion, this study presents 76 miRNAs with differential expression in the circulation of women with Luminal A-like breast cancer compared to those who do not have breast cancer. A miRNA profile of three circulating tumor-associated miRNA biomarkers (*miR-29a, miR-181a* and *miR-652)* for breast cancer are identified which in combination provide a sensitivity and specificity profile which exceeds that of several current clinical biomarkers. A complementary test, for use in combination with mammography would prove extremely advantageous particularly in an era where swift diagnosis, expeditious commencement of appropriate adjuvant treatments and surgical resection have a role to play in ultimately improving patient outcomes. Further large prospective studies are required, to include all breast cancer subtypes and to elucidate the potential of miRNAs in the systemic circulation as subtype-specific diagnostic or therapeutic breast cancer markers.

## Supporting Information

Figure S1 **Correlation between microarray and RQ-PCR data.** Correlation (Pearson’s) of miRNA expression levels between microarray (dark) and RQ-PCR (light) detected expression levels (A) *miR-29a* (B) *miR-181a* (C) *miR-182.*
(PNG)

Table S1 **MiRNAs with altered expression in Luminal A breast cancer.**
(DOC)
