# Supplementary figures and images for: Identification and Validation of Oncologic miRNA Biomarkers for Luminal A-like Breast Cancer
Source: PLoS One. 2014 Jan 31;9(1):e87032. doi: 10.1371/journal.pone.0087032 (PMC3909065; doi:10.1371/journal.pone.0087032)

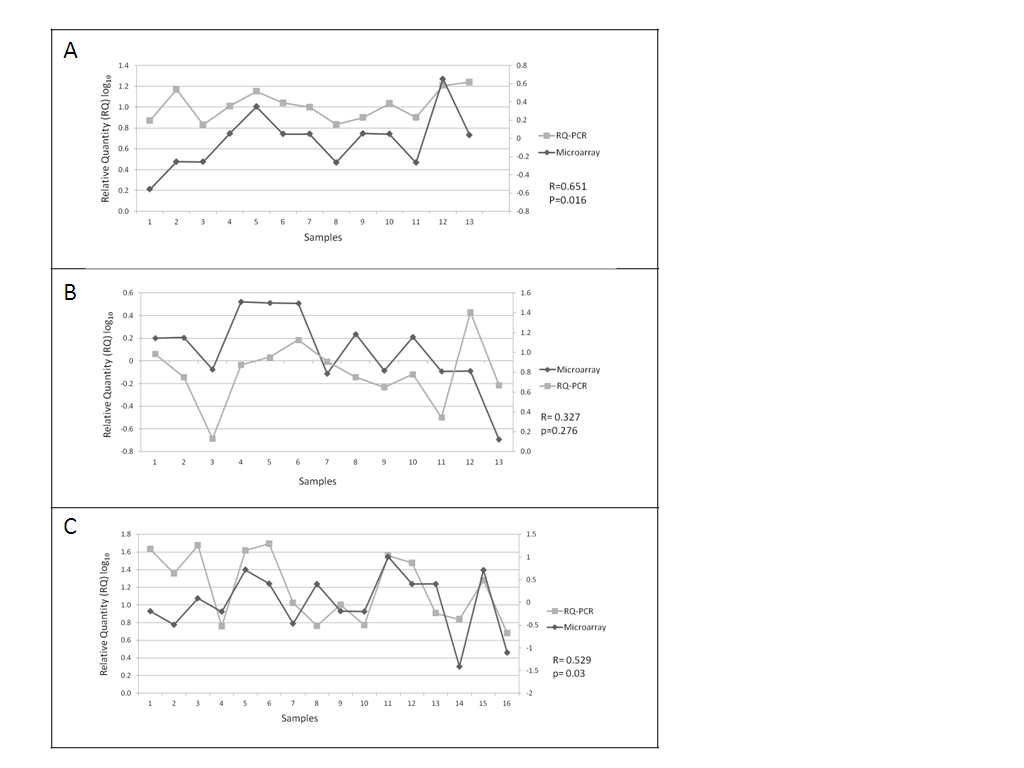

Supplement: Figure S1 — Correlation between microarray and RQ-PCR data. Correlation (Pearson’s) of miRNA expression levels between microarray (dark) and RQ-PCR (light) detected expression levels (A) miR-29a (B) miR-181a (C) miR-182. (PNG) [file pone.0087032.s001.png]
