# Supplementary material for: Identification and Validation of Oncologic miRNA Biomarkers for Luminal A-like Breast Cancer
Source: PLoS One. 2014 Jan 31;9(1):e87032. doi: 10.1371/journal.pone.0087032 (PMC3909065; doi:10.1371/journal.pone.0087032)
Supplement: Table S1 — MiRNAs with altered expression in Luminal A breast cancer. (DOC) [file pone.0087032.s002.doc]

Supplementary Table 1: MiRNAs with altered expression in Luminal A breast cancer

| **Rank** | **miRNA** |
| --- | --- |
| 1 | miR-181a |
| 2 | miR-301a |
| 3 | miR-182 |
| 4 | miR-423-5p |
| 5 | miR-19b |
| 6 | miR-93 |
| 7 | miR-652 |
| 8 | miR-29a |
| 9 | miR-486-5p |
| 10 | miR-223 |
| 11 | miR-532-5p |
| 12 | miR-30c |
| 13 | miR-103 |
| 14 | miR-339-3p |
| 15 | miR-183 |
| 16 | miR-320 |
| 17 | miR-29c |
| 18 | miR-210 |
| 19 | miR-425 |
| 20 | miR-125a-5p |
| 21 | miR-196b |
| 22 | miR-20b |
| 23 | miR-222 |
| 24 | miR-24 |
| 25 | miR-155 |
| 26 | miR-106a |
| 27 | miR-20a |
| 28 | miR-192 |
| 29 | miR-532-3p |
| 30 | miR-185 |
| 31 | miR-191 |
| 32 | miR-744 |
| 33 | let-7g |
| 34 | miR-30b |
| 35 | miR-324-3p |
| 36 | miR-660 |
| 37 | miR-25 |
| 38 | miR-454 |
| 39 | let-7c |
| 40 | let-7e |
| 41 | miR-345 |
| 42 | let-7d |
| 43 | miR-628-5p |
| 44 | let-7b |
| 45 | miR-16 |
| 46 | miR-17 |
| 47 | miR-331-3p |
| 48 | miR-886-5p |
| 49 | miR-501-5p |
| 50 | miR-106b |
| 51 | RNU48 |
| 52 | miR-146a |
| 53 | miR-15b |
| 54 | miR-126 |
| 55 | miR-146-5p |
| 56 | miR-374b |
| 57 | miR-19a |
| 58 | miR-194 |
| 59 | miR-200c |
| 60 | miR-374a |
| 61 | miR-186 |
| 62 | miR-28-3p |
| 63 | miR-342-3p |
| 64 | miR-92a |
| 65 | miR-145 |
| 66 | miR-140-3p |
| 67 | miR-26a |
| 68 | miR-142-3p |
| 69 | miR-150 |
| 70 | miR-451 |
| 71 | miR-26b |
| 72 | miR-486-5p |
| 73 | miR-574-3p |
| 74 | miR-139-5p |
| 75 | miR-195 |
| 76 | miR-484 |
